# Association of household food insecurity with developmental delay in preschool children: 2018 Ecuadorian Nutrition and Health National Survey

**DOI:** 10.1017/jns.2023.70

**Published:** 2023-08-01

**Authors:** M. Margaret Weigel, Rodrigo X. Armijos

**Affiliations:** 1 Department of Environmental & Occupational Health, Indiana University Bloomington, Bloomington, IN, USA; 2 Global Environmental Health Research Laboratory, Indiana University-Bloomington School of Public Health, Bloomington, IN, USA; 3 Center for Latin American and Caribbean Studies, Indiana University, Bloomington, IN, USA; 4 Indiana University Center for Global Health Equity, Indianapolis, IN, USA

**Keywords:** Early childhood developmental delay, Ecuador, Household food insecurity, Latin America, Preschool children

## Abstract

We investigated the association of household food insecurity (HFI) with developmental delays in 36–59-month-old preschool children (*n* 7005) using cross-sectional data from the 2018 Ecuadorian National Health and Nutrition Survey. HFI was assessed with the Food Insecurity Experience Scale and developmental delays with the Early Childhood Development Index. Log-binomial regression models estimated the association of HFI with global (overall) developmental delay (GDD) and delays in four individual developmental domains, adjusting for covariates. Nearly half of the children lived in households with marginal (24⋅5 %) or moderate-severe HFI (21⋅7 %). Eighteen percent were identified with GDD. Delays in the individual domains of literacy-numeracy, social-emotional, physical and cognitive development were identified for 64, 21⋅5, 3⋅3 and 3⋅1 %, respectively. GDD was more likely among preschool children from households with marginal (aPR = 1⋅29; 95 % C.I. = 1⋅10, 1⋅49) and moderate-severe HFI (aPR = 1⋅30; 95 % C.I. = 1⋅11, 1⋅51). Social-emotional development delays were also more likely among those from households with marginal (aPR = 1⋅36; 95 % C.I. = 1⋅19, 1⋅56) and moderate-severe HFI (aPR = 1⋅33; 95 % C.I. = 1⋅15, 1⋅54) different from the other three domains. Several other potentially modifiable risk (violent discipline, maternal depressive symptoms) and protective factors (adequate child stimulation, higher maternal education, handwashing with soap/detergent) were also independently associated with GDD and/or literacy-numeracy and cognitive delays. Our findings suggest that HFI is an independent risk factor for GDD and social-emotional developmental delays in Ecuadorian preschoolers. They underscore the importance of strengthening and expanding poverty reduction, food security and early childhood development policies and interventions to improve the opportunities for children to achieve their full developmental potential.

## Introduction

Early childhood developmental delay (ECDD) is a major global public health concern including in low- and middle-income countries (LMICs) where an estimated 250 million children under the age of five are at-risk for not achieving their full developmental potential^(1)^. ECDD occurs when a child does not achieve basic developmental milestones in cognitive, literacy-numeracy, physical and social-emotional domains at an expected age^(2)^. These delays can be individual or involve two or more domains (global developmental delay). The importance of ECDD is underscored by its inclusion as one of the UN 2030 Sustainable Development Goals (Goal 4⋅2) and its implicit role in several others^(3)^.

Early childhood development is affected by multiple biological, social and environmental determinants and their interactions across multiple levels of influence. Household-level exposures linked in the literature to ECDD include material poverty, environmental toxins, maternal mental illness, suboptimal parenting practices, violence, lack of adequate stimulation and learning opportunities, poor quality diets and malnutrition^(4–10)^. Another is exposure to household food insecurity (HFI) or ‘the limited or uncertain availability of households to obtain nutritionally adequate and safe foods or the limited or uncertain ability to acquire acceptable foods in socially acceptable ways’^(11)^.

Both nutritional and non-nutritional pathways have been proposed to explain how HFI may cause developmental delays in young children^(12–15)^. One of the nutritional pathways is through the adverse impact of HFI on dietary quality/quantity, child nutrition and health (e.g., micronutrient deficiencies, stunting, infections) and ultimately on neurological and physical development. Another potential nutritional pathway is through the negative effects of HFI on the dietary energy and micronutrient intakes of children as well as their parental caregivers. A consequence of this is that both children and their parents may be more likely to experience fatigue, lethargy and/or depressive symptoms. This can lead to decreased quality and quantity of parental nurturing, parent–child interactions and child interactions with other adults and children in the child's social sphere, which are all essential for normal child development. In addition, it has been hypothesised that feelings of stress, anxiety and depression experienced by parental caregivers over their household food security situation can negatively affect their child's nurturing behaviour and other interactions and ultimately, their child's development. Another hypothesised pathway is that the poverty-related financial constraints experienced by food-insecure households may make them less able to invest in early childhood education and physical resources (e.g., educational toys, books) that stimulate child development.

Research investigating the association of HFI with ECDD in preschool children is relatively limited compared to other child age groups. Most studies focused on the first 1000 d of life (age <3 years) because it is a period of rapid brain growth and development^(16)^. However, the second 1000 d of life (preschool period) is also important. This developmental period is characterised by the maturation of the anatomical and physiological substrates of the brain that is accompanied by increases in working memory, attention control, self-regulation and cognitive and social-emotional growth changes that set the stage for future academic, social and life success^(17,18)^. In preschool children, the failure to meet developmental milestones is strongly linked to reduced school readiness, decreased educational achievement, lower future economic productivity and income and the intergenerational transmission of poverty^(1,19,20)^.

Most prior studies examining the relationship between HFI and ECDD in preschoolers were conducted in the U.S. and other high-income countries (HIC), using different study designs, analytic methods and instruments. Their findings generally suggest that those living in food-insecure households were more likely to show evidence of delayed development, especially in the social-emotional domain, compared to those from fully food secure households^(12–14)^. Findings from the few studies published on this age group in LMICS have reported mixed results with respect to the association of HFI with social-emotional, cognitive or other specific developmental domains^(15,21)^. Moreover, none were conducted in LMICS in Latin America, a region where 30 % or more of all households suffer from moderate-severe HFI^(22,23)^ and global developmental delay is estimated to affect 25 % of all preschool children^(2)^. The investigation of this relationship in Latin American LMICs is important since not only does the prevalence of HFI and ECDD differ from HIC in the larger Americas region but they also frequently vary on household-level risk and protective factors as well as population access to early childhood development programs, social welfare, health care and child food and nutrition policies and programs. Understanding the relationship between exposure to HFI and developmental delays in preschool children is important for informing policy and interventions can reduce both and improve their future chances for better well-being, educational prospects and future productivity.

Ecuador is a middle-income South American country with high rates of income poverty (25 %) and extreme poverty (10⋅7 %)^(24)^ as well as moderate-severe HFI (32⋅7 %)^(23)^. The sole Ecuadorian study to report ECDD prevalence estimates for preschoolers noted that 73 % of the 48–61-month-old children studied had difficulties with problem-solving and 28 % had fine motor skill delay^(25)^. Although one Ecuadorian study linked HFI to psychosocial dysfunction and social-emotional delay (i.e., internalising and externalising behaviours) in 6–12-year-old public elementary school children, none have previously examined the relationship between HFI and ECDD in preschool-age children in this population^(26)^.

The present study analysed the data from the nationally representative 2018 Ecuadorian Nutrition and Health National Survey (ENSANUT) to examine the relationship of HFI with developmental delay in preschool children aged 36–59 months. Our working hypothesis was that HFI would be associated with global and domain-specific developmental delays in preschoolers due to its adverse effects on the nutrition and physical and/or psychological health of preschoolers and their maternal caregivers.

## Methods

### Study design and setting

We analysed cross-sectional data from the most recent 2018 Ecuadorian Nutrition and Health National Survey (ENSANUT) to examine the study hypothesis. The survey design, sampling method and other methodological characteristics have been previously described in detail in publicly available survey documents published by the Ecuadorian National Institute of Statistics and Censuses^(27,28)^. Briefly, the survey used a stratified multistage probabilistic sampling design to collect data during 2018–2019 from a nationally representative sample of households and children under the age of 5 years living in these households. The research universe consisted of all households and non-institutionalised adults and children living in households in the twenty-four Ecuadorian provinces. Most (80 %) of the survey data were collected from November 2018–January 2019 and the rest from June–July 2019^(28)^. All data were collected prior to the first confirmed COVID-19 case (29 February 2020) in Ecuador.

The sampling frame for the survey was drawn from 2010 to 2017 Ecuadorian population census data. In the first stage, a stratified sample of primary sampling units (PSU) was selected with the probability of selection proportional to the size of the Ecuadorian population. A total of 2590/2591 (99⋅96 %) of the selected PSUs were successfully interviewed. In the second stage, dwellings within each PSU were randomly selected. These were then were stratified into two groups: households having at least one child under the age of 5 years and households without any children under the age of 5 years. Of the 16 982 households selected with a child under the age of 5, 16 430 or 96⋅7 % were successfully interviewed by the survey team. Data - entered into the database were systematically evaluated by survey personnel to identify missing or improbable values and were also inspected for discrepancies against what had been recorded by field interviewers in the written interview documents. When any such problems were identified, the survey team contacted the respondent by telephone or returned to their household to obtain and/or verify the data in question. Fewer than 1⋅2 % of the responses in the ENSANUT 2018 survey were unable to be corrected or verified using these procedures^(27,28)^.

The present analysis used data from two modules in the 2018 ENSANUT survey, i.e., Child Development and Household Modules. Within the surveyed households containing one or more children under the age of 5 years, only one child per household aged 36–59 months was selected to participate in the Child Development module interviews in order to prevent household oversampling. The child in this age range whose birthday fell closest to the date (month and day) when their household was surveyed was selected for inclusion. The total number of preschool child cases potentially available for analysis was 9028. We sequentially excluded cases missing data on one or more of the four ECDI domain-specific questions (*n* 1727) or covariates (*n* 296). This resulted in 7005 cases being available for the final analyses. The publicly available data used in the study were fully de-identified^(28)^. For this reason, the Indiana University Institutional Review Board classifies the study as, ‘research not subject to human subject regulations.’

### Measures

#### Early childhood developmental delay

The Early Childhood Development Index (ECDI) was used by the survey to collect data on suspected developmental delays in preschool children. The ECDI is an international population-based screening measure developed by the United Nations/UNICEF for Multiple Indicator Cluster Surveys (MICS). The instrument was designed to capture the achievement of key developmental milestones in early childhood and facilitate comparisons across diverse populations^(29)^. As Table 1 shows, it consists of ten questions focused on milestones pertaining to four developmental domains: literacy-numeracy, physical, cognitive and social-emotional. Maternal caregivers were asked to report on whether their child met each of these. The three literacy-numeracy domain questions asked caregivers if their child could identify or name at least ten letters of the alphabet, read at least four simple, common words and knew the name of or could recognise the symbols of all numbers from 1 to 10. The two physical domain questions asked whether the child was able to pick up a small object with two fingers such as a stick or rock from the ground and whether they are sometimes too sick to play. The two cognitive domain questions questioned caregivers if their child is able to follow simple directions on how to do something correctly and when given something to do, they can do it independently. Finally, the three social-emotional domain questions asked whether the child gets along well with other children, whether they kick, bite or hit other children and whether they become easily distracted.

**Table 1.** Early childhood development index (ECDI) milestones and coding: domain-specific and global development^a^

	ECDI milestones	On-track development	Off-track development
**Domain-specific development**			
Social-emotional	Child gets along well with other children	Passed 2 or 3 milestones	Failed 1 or more milestones
	Child does not kick, bite or hit other children or adults		
	Child does not become easily distracted		
Cognitive	Child follows simple directions on how to do something correctly	Passed 1 or 2 milestones	Failed both milestones
	When child is given something to do, they are able to do it independently		
Literacy-numeracy	Child can identify or name at least 10 letters of the alphabet	Passed 2 or 3 milestones	Failed 1 or more milestones
	Child can read at least four simple, popular words		
	Child knows the name of and recognises the symbols of all numbers from 1 to 10		
Physical	Child can pick up a small object with two fingers like a stick or a rock from the ground	Passed 1 or 2 milestones	Failed both milestones
	Child is not sometimes too sick to play		
**Global development**		Passed 3 or 4 individual domain milestones	Failed 2 or more individual domain milestones

a Table modified from Bliznashka *et al.*^(6)^

As Table 1 indicates, the ECDI classifies children as being developmentally ‘on track’ if they pass at least one milestone in the physical and cognitive domains and at least two milestones in the social-emotional and literacy-numeracy domains^(29)^. For assessing global or overall development delays, children are considered ‘on track’ if they pass at least three of the four individual developmental domains. However, since our study was focused on the association of HFI with suspected developmental delays, we used developmentally ‘off-track’ as the outcome (Table 1). Thus, preschoolers in the study were classified as ‘off track’ if they failed both milestones in the physical and cognitive domains and failed one or more of those in the social-emotional and literacy-numeracy domains. Children who did not achieve the benchmarks for two or more individual domains were classified with suspected global developmental delay (Table 1)**.**

### Household food insecurity

HFI was the main exposure variable in this study. The food security situation of households was assessed using the Food Insecurity Experience Scale (FIES)^(30)^. The FIES includes eight ‘yes/no’ questions focused on the self-reported, food-related behaviours and experiences of households in the previous 12 months that were associated with difficulties in accessing food resulting from resource constraints^(30)^. Affirmative responses by the maternal caregiver or other household head to the eight items were summed to produce a raw score (range 0–8) with higher scores indicating more severe food insecurity. Households who reported no affirmative responses were classified as food secure, those with 1–3 affirmative responses as marginally food insecure and those with 4–8 affirmative responses with moderate-severe food insecurity.

### Covariates

Child, maternal caregiver and household variables available in the ENSANUT survey database were considered for potential inclusion in the statistical models based on their reported theoretical or reported associations with ECDI and HFI. These were child age (36–47 months, 48–59 months), gender (female, male) and adequate stimulation exposure (yes, no). The survey assessed child exposure to adequate stimulation using the Demographic Health Surveys (DHS) home stimulation module questions. This instrument collected data from maternal caregivers on whether the child had been provided with six specific types of stimulation activities in the prior 3 d by any household adults^(31)^. These activities included reading books or looking at pictures, telling stories, naming, counting, drawing, singing, taking the child outside and/or playing with the child. The total number of possible positive responses on the instrument ranged from 0 to 6. Adequate stimulation was defined as the provision of four or more stimulation activities to the child^(32)^.

Exposure to violent disciplinary measures was measured in the survey using items from the UNICEF-modified version of the Parent–Child Conflict Tactics Scale^(33)^. This instrument questioned maternal caregivers about whether they or any other household adults had subjected the child to any sort of violent physical or psychological disciplinary practices in the prior 30 d. The six yes/no questions on the physical aggression subscale included shook the child, spanked the child, hit or slapped the child on the arm or leg, hit the child with an object, hit or slapped the child on the face, head or ears and beat the child as hard as one could. The two questions on the psychological aggression subscale (yes/no) were that they or another household adult shouted at the child or called them names. If the child was reported to have experienced any type of physically or psychologically violent discipline during the prior 30-d period, they were recorded as positive for exposure to violent discipline^(33)^.

Maternal covariates considered for inclusion included maternal ethnoracial minority (indigenous, afro-descendant) *v*. mestizo majority/other), education (none/basic education/primary school, secondary school or higher education) and depressive symptoms in maternal caregivers which were assessed using the Patient Health Questionnaire-8 (PHQ-8)^(34)^. The PHQ-8 was scored from 0 to 24, with higher scores indicating greater depressive symptoms. Maternal caregivers reporting a positive score of ≥10 on the scale were classified as positive for moderate to severe depressive symptoms^(35)^. Two household-level covariates considered for potential inclusion were region of residence (Andean highlands region, Pacific coastal region, Amazonian region, Galapagos insular region) and handwashing behaviour. The UN/UNICEF Joint Monitoring Programme for Water Supply, Sanitation and Hygiene (JMP) criteria were used to classify the reported handwashing behaviour of household members, i.e., soap or detergent used (i.e., bar or liquid soap/powder/liquid/paste detergent) *v.* water only or other (i.e., ash/clay/sand)^(36)^.

### Data analysis

We conducted descriptive analyses of the outcomes (global and domain-specific developmental delays), independent variables (HFI) and covariates. We used log-binomial regression analysis to estimate the association of HFI with global and domain-specific developmental delays. The findings from these analyses are presented as unadjusted and adjusted prevalence ratios (PR) with their respective 95 % confidence intervals (CI). For the adjusted models, covariates associated with HFI or global or domain-specific developmental delay of *P* < 0⋅10 were selected for inclusion. These were entered simultaneously (forced entry) in the models. We investigated potential multicollinearity by examining the variance of inflation factors (VIF) and tolerance. VIF values ranged between 1⋅01 and 1⋅09 and tolerance values ranged between 0⋅92 and 1⋅0 suggesting that multicollinearity was not a significant issue^(37)^. The models were also examined for potential interaction effects but no meaningful effect modifications were identified. We used survey-provided sample weights for the analyses to account for the complex design of the 2018 ENSANUT national survey^(28)^. The data were analysed using Stata (StataCorp. 2021. *Stata Statistical Software: Release 17*. College Station, TX: StataCorp LLC. All statistical tests were two-tailed.

## Results

### Sample characteristics

Table 2 displays the household food security situation and other characteristics of the Ecuadorian preschool children in the study. Close to half of all the households were affected by marginal (24⋅5 %) or moderate-severe food insecurity (21⋅7 %). Slightly more than half of the children were between the ages of 48 and 59 months and were male. A majority were reported to have received adequate stimulation in the home from maternal caregivers and/or other household adults (79 %). Most (70 %) also were reported to have been disciplined by household adults using violent psychological or physical measures. Maternal caregivers were predominantly from the mestizo ethnoracial majority group (84 %), two-thirds had at least some secondary or higher formal education (66 %) and 5 % reported PHQ-8 symptom scores of ≥10 suggesting possible moderate-severe depression. Two-thirds of the preschoolers resided in households located in the provinces in the Andean highlands (34 %) or the Pacific coastal plain region (34 %) with the remainder in Ecuadorian Amazonian provinces (29 %) or the Galapagos Islands (3 %). Most households reported using soap or detergent (90 %) for handwashing rather than water only or another substance such as ash, clay or sand.

**Table 2.** Child, maternal and household characteristics (*n* 7005)

	Unweighted No.	Weighted %
**Household food security status**		
Food secure	3833	53⋅8
Marginal food insecurity	1695	24⋅5
Moderate-severe food insecurity	1477	21⋅7
**Child age**		
36–47 months	3460	48⋅9
48–59 months	3545	51⋅1
**Child gender**		
Female	3408	48⋅7
Male	3597	51⋅3
**Receives adequate stimulation provided by caregivers**		
Yes	5546	78⋅6
No	1459	21⋅4
**Exposure to violent disciplinary measures**		
Yes	4926	70⋅1
No	2079	29⋅9
**Maternal ethnoracial group**		
Minority group	1093	15⋅6
Mestizo/other group	5912	84⋅4
**Maternal education**		
None/basic education/primary school	2371	34⋅2
Secondary school or higher	4634	65⋅8
**Maternal depressive symptoms (PHQ-8)**		
Yes (score ≥10)	347	4⋅7
No (score <10)	6658	95⋅3
**Household geographical region**		
Andean highlands region	2793	34⋅2
Pacific coastal region	2605	34⋅1
Amazon region	1463	28⋅6
Galapagos insular region	144	3⋅1
**Household handwashing with soap or detergent**		
Yes	6227	89⋅2
No	772	10⋅8

### Early childhood developmental delay

Based on their ECDI scores, 64 % of the preschool children were identified as being off-track in literacy-numeracy development, 21⋅5 % in social-emotional development, 3⋅3 % in physical development and 3⋅1 % in cognitive development. Eighteen percent were identified with global (overall) developmental delay (GDD) as they were off-track in two or more individual domains and 28 % showed no evidence of delay in any of the four developmental domains.

Table 3 displays the unadjusted findings from the log-binomial regression analyses examining the association of HFI with GDD and domain-specific development delay. Preschoolers living in households with marginal or moderate-severe HFI were 1⋅42 and 1⋅51 times more likely, respectively, than children from the food secure reference group to be identified with GDD. The table also shows findings from the analyses examining the association of HFI with delays in the four individual developmental domains. Children from households with either marginal or moderate HFI were about 1⋅5 times more likely than those from the reference group to show evidence of delayed social-emotional development. Those from households with moderate-severe but not marginal HFI were 1⋅10, 1⋅40 and 1⋅42 times more likely, respectively, to show evidence of delays in literacy-numeracy, physical or cognitive development compared to reference group children.

**Table 3.** Unadjusted association of HFI with off-track domain-specific and global development (*n* 7005)

	Off-track: global development		Domain-specific developmental delays							
			Any off-track: literacy-numeracy domain		Any off-track: physical domain		Off-track: cognitive		Off-track: social-emotional domain	
	%	PR (95 % CI)	%	PR (95 % CI)	%	PR (95 % CI)	%	PR (95 % CI)	%	PR (95 % CI)
**Household food security status**										
Food secure	14⋅5	1⋅00 (ref.)	62⋅1	1⋅00 (ref.)	2⋅8	1⋅00 (ref.)	2⋅5	1⋅00 (ref.)	17⋅9	1⋅00 (ref.)
Marginal food insecurity	20⋅6	1⋅42 (1⋅23, 1⋅63)^a^	63⋅5	1⋅05 (0⋅96, 1⋅15)	4⋅2	1⋅31 (0⋅97, 1⋅77)	2⋅8	0⋅94 (0⋅66, 1⋅34)	25⋅7	1⋅45 (1⋅27, 1⋅65)^a^
Moderate-severe food insecurity	21⋅9	1⋅51 (1⋅30, 1⋅75)^a^	67⋅9	1⋅10 (1⋅02, 1⋅21)^c^	3⋅9	1⋅40 (1⋅02, 1⋅90)^b^	4⋅9	1⋅42 (1⋅03, 1⋅97)^b^	25⋅6	1⋅46 (1⋅27, 1⋅68)^a^

PR,  prevalence ratio; %, weighted percent.

a *P* = 0⋅001.

b *P* = 0⋅03.

c *P* = 0⋅04, all other *P*-values > 0⋅05.

In addition to HFI, certain child, maternal and household characteristics were associated with developmental delays among the Ecuadorian preschoolers in the study. As Table 4 shows, younger children (36–47 months) were more likely than older ones (48–59 months) to be identified with GDD and delayed literacy-numeracy and social-emotional development. Male children were more likely to have GDD and delayed social-emotional development. Those reported as having adequate stimulation were less likely than those without it to be identified with GDD and developmental delays in all four individual domains. Children disciplined by household adults using physical or psychological violence were more likely to be identified with GDD and individual delays in the social-emotional and literacy-numeracy domains. The unadjusted findings also suggested that children with better-educated maternal caregivers were less likely to be identified with GDD and with domain-specific delays in literacy-numeracy, cognitive or social-emotional development compared to those with more poorly educated caregivers. Children whose maternal caregivers self-identified as an ethnoracial minority (indigenous, afro-descendant) were more likely to be classified with GDD and with delayed cognitive development.

**Table 4.** Unadjusted association of other child, maternal and household characteristics with off-track domain-specific and global development (*n* 7005)

			Domain-specific developmental delays							
	Off-track: global		Off-track: literacy-numeracy domain		Off-track: physical domain		Off-track: cognitive		Off-track: social-emotional	
Characteristics	%	PR (95 % CI)	%	PR (95 % CI)	%	PR (95 % CI)	%	PR (95 % CI)	%	PR (95 % CI)
**Child age**										
36–47 months	20⋅5	1⋅36 (1⋅21, 1⋅54)^a^	70⋅1	1⋅23 (1⋅14, 1⋅33)^a^	3⋅5	1⋅18 (0⋅92, 1⋅52)	3⋅2	1⋅20 (0⋅91, 1⋅58)	22⋅9	1⋅13 (1⋅01, 1⋅26)^b^
48–59 months	14⋅9	1⋅00 (ref.)	57⋅5	1⋅00 (ref.)	3⋅1	1⋅00 (ref.)	3⋅0	1⋅00 (ref.)	20⋅1	1⋅00 (ref.)
**Child gender**										
Female	15⋅7	1⋅00 (ref.)	63⋅5	1⋅00 (ref.)	3⋅5	1⋅00 (ref.)	3⋅2	1⋅00 (ref.)	18⋅8	1⋅00 (ref.)
Male	19⋅5	1⋅27 (1⋅12, 1⋅43)^a^	63⋅8	1⋅02 (0⋅94, 1⋅10)	3⋅2	1⋅11 (0⋅86, 1⋅43)	3⋅0	0⋅95 (0⋅72, 1⋅25)	24⋅0	1⋅28 (1⋅14, 1⋅43)^a^
**Adequate child stimulation**										
Yes	15⋅8	0⋅63 (0⋅55, 0⋅72)^a^	61⋅3	0⋅84 (0⋅77, 0⋅92)^a^	3⋅2	0⋅70 (0⋅53, 0⋅93)^d^	2⋅3	0⋅46 (0⋅34, 0⋅61)^a^	20⋅6	0⋅80 (0⋅70, 0⋅91)^a^
No	24⋅4	1⋅00 (ref.)	72⋅5	1⋅00 (ref.)	3⋅9	1⋅00 (ref.)	5⋅9	1⋅00 (ref.)	24⋅7	1⋅00 (ref.)
**Violent child discipline**										
Yes	19⋅6	1⋅50 (1⋅30, 1⋅73)^a^	65⋅5	1⋅11 (1⋅02, 1⋅20)^c^	3⋅4	1⋅04 (0⋅79, 1⋅37)	2⋅9	0⋅80 (0⋅60, 1⋅07)	23⋅7	1⋅51 (1⋅32, 1⋅72)^a^
No	13⋅1	1⋅00 (ref.)	59⋅3	1⋅00 (ref.)	3⋅2	1⋅00 (ref.)	3⋅6	1⋅00 (ref.)	16⋅1	1⋅00 (ref.)
**Maternal education**										
Secondary school or higher education	21⋅2	0⋅70 (0⋅62, 0⋅80)^a^	69⋅3	0⋅87 (0⋅80, 0⋅94)^a^	3⋅6	0⋅78 (0⋅60, 1⋅01)	4⋅5	0⋅48 (0⋅37, 0⋅64)^a^	24⋅2	0⋅84 (0⋅75, 0⋅94)^e^
None/basic education/primary school only	15⋅4	1⋅00 (ref.)	60⋅8	1⋅00 (ref.)	3⋅1	1⋅00 (ref.)	2⋅3	1⋅00 (ref.)	19⋅6	1⋅00 (ref.)
**Maternal ethnoracial group**										
Minority group	20⋅4	1⋅23 (1⋅05, 1⋅44)^e^	63⋅6	1⋅01 (0⋅91, 1⋅12)	3⋅7	1⋅21 (0⋅87, 1⋅67)	5⋅4	2⋅35 (1⋅74, 3⋅18)^a^	23⋅1	1⋅14 (0⋅99, 1⋅32)
Mestizo/other group	17⋅1	1⋅00 (ref.)	63⋅7	1⋅00 (ref.)	3⋅3	1⋅00 (ref.)	2⋅7	1⋅00 (ref.)	21⋅2	1⋅00 (ref.)
**Maternal PHQ-8 score**										
Score ≥10	27⋅3	1⋅57 (1⋅24, 1⋅99)^a^	63⋅4	1⋅07 (0⋅91, 1⋅27)	3⋅5	1⋅13 (0⋅65, 1⋅96)	3⋅1	1⋅18 (0⋅65, 2⋅13)	20⋅8	1⋅68 (1⋅36, 2⋅08)^a^
Score <10	17⋅2	1⋅00 (ref.)	68⋅2	1⋅00 (ref.)	3⋅3	1⋅00 (ref.)	3⋅2	1⋅00 (ref.)	35⋅5	1⋅00 (ref.)
**Household region**										
Andean highlands	2⋅7	1⋅00 (ref.)	63⋅3	1⋅00 (ref.)	3⋅1	1⋅00 (ref.)	21⋅6	1⋅00 (ref.)	16⋅6	1⋅00 (ref.)
Pacific coast	2⋅8	0⋅99 (0⋅86, 1⋅13)	65⋅1	0⋅99 (0⋅91, 1⋅07)	3⋅1	0⋅89 (0⋅67, 1⋅19)	21⋅9	0⋅86 (0⋅62, 1⋅20)	18⋅0	0⋅97 (0⋅86, 1⋅10)
Amazon	4⋅0	1⋅04 (0⋅89, 1⋅22)	63⋅1	0⋅97 (0⋅88, 1⋅07)	3⋅9	1⋅02 (0⋅73, 1⋅42)	21⋅3	1⋅41 (1⋅02, 1⋅99)	19⋅0	0⋅96 (0⋅83, 1⋅12)
Galapagos	1⋅4	0⋅73 (0⋅45, 1⋅18)	57⋅6	0⋅89 (0⋅67, 1⋅17)	2⋅8	0⋅74 (0⋅27, 2⋅03)	17⋅4	0⋅48 (0⋅12, 1⋅97)	13⋅2	0⋅78 (0⋅51, 1⋅21)
**Household uses soap or detergent for handwashing**										
Yes	16⋅0	0⋅71 (0⋅60, 0⋅85)^a^	60⋅2	0⋅88 (0⋅78, 0⋅99)^b^	3⋅2	0⋅93 (0⋅63, 1⋅36)	2⋅4	0⋅39 (0⋅28, 0⋅53)^a^	20⋅3	0⋅83 (0⋅70, 0⋅98)^b^
No	19⋅7	1⋅00 (ref.)	68⋅1	1⋅00 (ref.)	3⋅5	1⋅00 (ref.)	3⋅9	1⋅00 (ref.)	23⋅0	1⋅00 (ref.)

PR , prevalence ratio; %, weighted percent.

a *P* = 0⋅001.

b *P* = 0⋅03.

c *P* = 0⋅01.

d *P* = 0⋅04.

e *P* = 0⋅002, all other *P*-values > 0⋅05.

The analyses also showed that GDD and delayed social-emotional development were more prevalent in the children whose maternal caregivers reported greater depressive symptoms. Children from households reporting the use of soap/detergent for handwashing rather than plain water or another substance were less likely to be identified with GDD or domain-specific delays in literacy-numeracy, cognitive or social-emotional development. In contrast, the region where a child lived was not associated with GDD nor any individual developmental delay.

Table 5 displays the findings from the adjusted log-binomial regression models suggesting that HFI was a major independent risk factor for global or overall developmental delay and one domain-specific delay in the Ecuadorian preschool children. Specifically, preschoolers from households having either marginal or moderate-severe HFI were 1⋅3–1⋅4 times more likely than those from food-secure homes to be identified with GDD or delayed social-emotional development after adjusting for model covariates. In addition to HFI, the adjusted analyses also identified certain other risk and protective factors independently associated with GDD and social-emotional developmental delay. Risk factors for these included younger age, being male, exposure to violent discipline and greater maternal caregiver depressive symptoms. In contrast, receiving adequate stimulation from household adults appeared protective against GDD and social-emotional developmental delay. Having a better-educated maternal caregiver also was associated with reduced GDD-.

**Table 5.** Adjusted association of HFI and other child, maternal and household characteristics with off-track global and domain-specific child development indicators (*n* 7005)

	Off-track: global	Domain-specific developmental delays			
		Off-track: literacy-numeracy domain	Off-track: physical domain	Off-track: Cognitive domain	Off-track: social-emotional domain
	aPR (95 % CI)	aPR (95 % CI)	aPR (95 % CI)	aPR (95 % CI)	aPR (95 % CI)
**Household food security**					
Food secure	1⋅00 (ref.)	1⋅00 (ref.)	1⋅00 (ref.)	1⋅00 (ref.)	1⋅00 (ref.)
Marginal food insecurity	1⋅29 (1⋅11, 1⋅49)^a^	1⋅02 (0⋅93, 1⋅11)	1⋅26 (0⋅93, 1⋅71)	0⋅80 (0⋅56, 1⋅15)	1⋅36 (1⋅19, 1⋅56)^a^
Moderate-severe food insecurity	1⋅30 (1⋅11, 1⋅51)^a^	1⋅05 (0⋅95, 1⋅16)	1⋅29 (0⋅93, 1⋅78)	0⋅96 (0⋅68, 1⋅36)	1⋅33 (1⋅15, 1⋅54)^a^
**Child age**					
36–47 months	1⋅38 (1⋅22, 1⋅56)^a^	1⋅24 (1⋅15, 1⋅33)^a^	1⋅18 (0⋅92, 1⋅52)	1⋅19 (0⋅90, 1⋅57)	1⋅14 (1⋅02, 1⋅28)^b^
48–59 months	1⋅00 (ref.)	1⋅00 (ref.)	1⋅00 (ref.)	1⋅00 (ref.)	1⋅00 (ref.)
**Child gender**					
Female	1⋅00 (ref.)	1⋅00 (ref.)	1⋅00 (ref.)	1⋅00 (ref.)	1⋅00 (ref.)
Male	1⋅24 (1⋅10, 1⋅40)^a^	1⋅01 (0⋅94, 1⋅09)	1⋅10 (0⋅86, 1⋅42)	0⋅93 (0⋅71, 1⋅23)	1⋅25 (1⋅12, 1⋅40)^a^
**Adequate child stimulation (≥4 activities)**					
Yes	0⋅72 (0⋅62, 0⋅83)^a^	0⋅87 (0⋅80, 0⋅96)^c^	0⋅76 (0⋅56, 1⋅01)	0⋅62 (0⋅45, 0⋅89)^a^	0⋅87 (0⋅76, 0⋅99)^d^
No	1⋅00 (ref.)	1⋅00 (ref.)	1⋅00 (ref.)	1⋅00 (ref.)	1⋅00 (ref.)
**Violent child discipline**					
Yes	1⋅45 (1⋅26, 1⋅67)^a^	1⋅11 (1⋅02, 1⋅21)^e^	1⋅01 (0⋅76, 1⋅33)	0⋅79 (0⋅59, 1⋅06)	1⋅44 (1⋅26, 1⋅65)^a^
No	1⋅00 (ref.)	1⋅00 (ref.)	1⋅00 (ref.)	1⋅00 (ref.)	1⋅00 (ref.)
**Maternal depressive symptoms**					
Yes (score ≥10)	1⋅38 (1⋅09, 1⋅75)^g^^f^	1⋅04 (0⋅60, 1⋅83)	1⋅05 (0⋅61, 1⋅84)	1⋅06 (0⋅58, 1⋅94)	1⋅50 (1⋅20, 1⋅86)^a^
No (score <10)	1⋅00 (ref.)	1⋅00 (ref.)	1⋅00 (ref.)	1⋅00 (ref.)	1⋅00 (ref.)
**Maternal education**					
Secondary school or higher education	0⋅80 (0⋅70, 0⋅91)^a^	0⋅89 (0⋅82, 0⋅97)^g^	0⋅86 (0⋅65, 1⋅12)	0⋅62 (0⋅46, 0⋅83)^a^	0⋅92 (0⋅81, 1⋅04)
None/basic education/primary school only	1⋅00 (ref.)	1⋅00 (ref.)	1⋅00 (ref.)	1⋅00 (ref.)	1⋅00 (ref.)
**Maternal ethnoracial group**					
Minority group	1⋅01 (0⋅86, 1⋅19)	0⋅93 (0⋅84, 1⋅04)	1⋅07 (0⋅76, 1⋅50)	1⋅70 (1⋅22, 2⋅36)^h^	1⋅02 (0⋅87, 1⋅19)
Mestizo/other group	1⋅00 (ref.)	1⋅00 (ref.)	1⋅00 (ref.)	1⋅00 (ref.)	1⋅00 (ref.)
**Household uses soap or detergent for handwashing**					
Yes	0⋅87 (0⋅72, 1⋅04)	0⋅92 (0⋅83, 1⋅04)	1⋅09 (0⋅73, 1⋅63)	0⋅54 (0⋅38, 0⋅77)^a^	0⋅95 (0⋅80, 1⋅13)
No	1⋅00 (ref.)	1⋅00 (ref.)	1⋅00 (ref.)	1⋅00 (ref.)	1⋅00 (ref.)

aPR, adjusted prevalence ratio.

a *P* = 0⋅001.

b *P* = 0⋅03.

c *P* = 0⋅005.

d *P* = 0⋅002.

e *P* = 0⋅05.

f *P* = 0⋅02.

g *P* = 0⋅009, all other *P*-values > 0⋅05.

h *P* = 0⋅007.

The findings also indicated that after adjustment for model covariates, the previously identified associations of HFI with literacy-numeracy, physical and cognitive developmental delays were no longer evident. However, certain child, maternal and/or household-level factors were independently associated in the adjusted analyses with delays in literacy-numeracy and cognitive development but not physical development. Risk factors for delayed literacy-numeracy development included younger child age, exposure to violent disciplinary measures and self-reported maternal minority group membership. In contrast, adequate stimulation and higher maternal caregiver education appeared protective. Adequate child simulation, higher maternal education and household handwashing with soap/detergent appeared protective against delayed cognitive development.

## Discussion

This study is the first to report on the relationship between HFI and developmental delays in either Ecuadorian or other Latin American preschool child populations. It furthers our understanding of the influence of HFI on development during the second 1000 d of life and adds to the limited studies published on this important topic for preschool children living in LMICs. The 22 % prevalence of moderate-severe HFI identified for the child households is consistent with pre-pandemic estimates recorded for Ecuador and other LMICs in the Latin America region^(23)^. The 18 % prevalence of suspected global developmental delay identified for the Ecuadorian preschoolers in this study was within the 11⋅4–18⋅9 % range recorded by other pre-covid pandemic national surveys for the same age group in other Latin American LMICs using the same assessment tool^(2)^. Likewise, the prevalence of reported delays in the four individual developmental domains was also similar^(2)^.

The study findings suggest that marginal and as well as moderate-severe HFI is independently associated with overall or global delays in development and, in particular, delays in the social-emotional development of preschool children. These findings underscore the importance of reducing exposure to any level of HFI during this important life course period. Our findings align with many, but not all, prior studies reporting on the association of HFI with ECDD in preschoolers in high-income populations^(37–43)^. They are also partially consistent with those reported by the two studies published on the relationship of HFI with social-emotional development indicators in preschool children from LMICs in other regions. One of these longitudinal studies reported that exposure to HFI was associated with greater externalising behaviour, an indicator of delayed social-emotional development in 4-year-old Pakistani children^(21)^. The other reported that although HFI was not associated with overall social-emotional development in Ghanese preschoolers, exposure to transitory HFI was marginally associated with poorer self-regulation, another social-emotional development indicator^(15)^.

We surmise that HFI was associated with social-emotional and global developmental delays in the Ecuadorian preschool children due to its direct effects on child diet, nutrition and psychosocial pathways and/or indirect effects on that of maternal caregivers. A number of studies have linked HFI with lower dietary quality^(44–47)^ and poorer nutritional status manifested as stunting^(47,48)^ and nutritional anaemia in children^(49,50)^ Such undernutrition can have harmful effects on the brain structures and processes of young children which manifests as poorer self-regulation and negative social behaviour^(16,38,51)^. In addition, parents are not always able to shield children from HFI^(52,53)^. Meal skipping, portion size reductions and other coping strategies used by food-insecure households can cause hunger pangs and reduced blood glucose levels in children which promote increased irritability, distractedness and/or fatigue. In addition, feeling hunger pangs has been linked to behaviours in children such as a reluctance to share or engage in fair play^(54)^. For these reasons, food insecure children may be less likely to engage in positive interactions with their caregivers, siblings or other adults and children. Furthermore, children living in food-insecure households are reported to often be aware of and stressed out by their home food situation^(55)^. The effects of this stress and the negative behavioural manifestations it may engender could also reduce the likelihood of positive social interactions of children with their caregivers, siblings and others in their households, at school and other social networks.

HFI may also indirectly affect overall development and social-emotional development in particular through its adverse effects on maternal caregivers. For example, maternal caregivers in food-insecure households often experience stress caused by concerns over being able to provide sufficient food for their children, other household members and themselves which can increase anxiety and depressive symptoms in vulnerable individuals. HFI has been linked to ethnospecific illnesses (nervousness, irritability, angry outbursts)^(56)^ and depressive symptoms in Ecuadorian maternal caregivers^(56–58)^. In addition, similar to children, insufficient caloric intake, meal skipping and other disordered eating patterns by adults in food-insecure households may promote irritability, distractibility and fatigue. Likewise, both hunger and low glucose levels are associated with negative emotionality in adults, i.e., increased impulsivity, anger, aggression^(59,60)^, which could cause maternal and other caregivers to be less responsive to the needs of their child and reduce positive parenting behaviours. Inadequate stimulation, less frequent positive interactions and low attachment by caregivers are well documented to have a negative impact on child development^(16,38,61,62)^. Another potential mechanism involves the effects of HFI on the micronutrient status of maternal caregivers. HFI has been linked to lower intakes and/or expenditures on micronutrient-rich foods and anaemia in maternal caregivers and other reproductive-age women in Ecuador^(63,64)^ and other LMICs^(65,66)^. Women with anaemia are more likely to suffer from depression, anxiety, other mental health disorders and sleep disturbances^(67)^ which also can impede the ability of maternal to positively interact with, stimulate or otherwise care for their child.

Different from social-emotional development, HFI was not associated with delayed literacy-numeracy and cognitive development in the Ecuadorian preschool children consistent with the mixed findings published on this topic in high-income^(12,13,20)^ and LMIC populations^(15,21)^. However, we did identify several other potentially modifiable household-level risk and protective factors associated with literacy-numeracy and/or cognitive developmental delays such as exposure to adequate stimulation or violent disciplinary measures, maternal education and handwashing behaviour. The reason for the lack of association of HFI or other independent risk or protective factors with physical developmental delay is uncertain but could be because this indicator is less susceptible to environmental influences compared to the three other developmental domains^(12)^.

### Study strengths and limitations

This study had certain strengths and limitations that should be considered when interpreting its findings. Some strengths included a nationally representative sample with a large sample size, a low non-response rate (1⋅2 %) and the use of standardised data collection procedures and quality control methods^(28)^. The Early Child Development Index (ECDI) is a widely used brief screener used to estimate the prevalence of suspected developmental delay in child populations. It has been validated in national surveys including in Latin American LMICs^(29)^. Likewise, the PHQ-8 is a well-validated scale used to screen for depressive symptoms in Latin American and other diverse populations^(35,68)^. Another strength includes the use of multivariable analysis to adjust for child stimulation, violent disciplinary measures, maternal depressive symptoms and other factors known to influence ECDD as well as a novel factor, i.e., household handwashing behaviour, which has been linked to both ECD^(69)^ and HFI^(70)^ but not examined in prior studies investigating the HFI-ECDD relationship.

One of the limitations was that the measurement of food security was restricted to the previous 12-month period, so it might not necessarily reflect the food situations of households over a longer period of time. Different persons living in the same household may experience different levels of food insecurity such as young children whom adults may try to protect from its consequences. Another limitation is that maternal caregivers were the source of information on child variables so reporting bias could have influenced the study results. It is also possible that maternal caregivers with higher levels of depressive symptoms or other mental health issues could make them more inclined to report socio-emotional and/or other developmental domain delays in their children. Moreover, while HFI may increase the risk for ECD through child and maternal mental health pathways, it is also possible that the relationship is bidirectional whereby these could also promote food insecurity in households. In this study, HFI was an important but not the only factor identified as influencing global development and three domain-specific delays. While we included a number of known confounders in our multivariable analysis, others were not because they were not contained in the survey database such as child and maternal micronutrient deficiencies and exposure to environmental toxicants known to adversely impact child development. Both nutritional anemias^(22,27)^ and high blood levels of lead and other neurotoxicants^(71)^ are reported as highly prevalent among children and reproductive-age women in Ecuador. Future studies should include these factors when assessing the HFI-developmental delay relationship in preschoolers. Finally, while the findings allow for inference about the HFI-developmental delay relationship, the study's cross-sectional survey design does not permit establishing causal or temporal effects.

## Conclusion

The preschool years represent a critical period of early child development that sets the stage for future success in social relationships, education and employment and other important life aspects. Our study findings suggest that HFI is an independent risk factor for global and social-emotional developmental delay in Ecuadorian preschool children. However, several other potentially modifiable household-level risk and protective factors were also associated with developmental delay in this population. In conclusion, our findings underscore the importance of strengthening and expanding existing national poverty reduction, food security and ECDD policies and interventions to improve the child opportunities for achieving their full developmental potential.
